# Clinical Introductory

**Published:** 1837

**Authors:** John P. Harrison

**Affiliations:** Professor of Mat. Med. and Pharmacy, in the Cincinnati College


### Art. II.—Clinical Introductory.

*The Indications of Cure in Diseases*: *An Introductory Lecture, to a Course of Clinical Instruction in the* Cincinnati Hospital. By John P. Harrison, M. D., Professor of Mat. Med. and Pharmacy, in the Cincinnati College.

Gentlemen:—I shall begin my course of Clinical Instruction in the wards of this Hospital, by delivering to you my views, in reference to the true curative indications, which should govern us in the administration of remedies. And it affords me much pleasure to meet you on this occasion, and to have this favorable opportunity of expressing to you my conceptions of general pathology, and the just and legitimate grounds upon which all rational medical interference in disease is to be established. Here is the great terminating point of all our acquisitions in medical science—it is at the foot of sound clinical practice that all our principles and facts, our dogmas and data, are to be laid. For the re-establishment of our patient's health—for the restoration of his normal physiological state, we toil at the preliminary studies of our science, and expend our painful researches into the records of medical erudition. In order to become good, safe, and efficient practitioners of medicine, we bend over the mangled *cadaver*, and dissect fibre after fibre, and explore cavity after cavity, and pursue nerve after nerve, and vessel after vessel, till the whole complex structure of man is revealed to our sight.

To cure the sick, we open and dissect the dead; to relieve the painful maladies incident to the human economy, we rifle nature of her secret articles of potent activity, and subject them to various processes to make them yield up their medicinal virtues. Anatomy, physiology, chemistry, botany; these and other indispensable elementary branches of study, all stand with eager and obedient willingness, to minister their lights, and pour in their contributions, to this great central point of all our efforts.

In yonder edifice, the science of medicine is taught by my colleagues and myself. In the lecture-rooms of the Cincinnati College, we endeavor, during four months of the year, to teach with minute precision, copious illustration, and instructive emphasis, the truths and doctrines—the facts and principles of the science of medicine. And, gentlemen, not satisfied with the ordinary mode of inculcating the truths of medical science by prelections, the

Faculty of the school have, as you are aware, opened this asylum for the sick, that suffering humanity might not only receive such aid at our hands, as the exigent condition of the sick might demand, but likewise, and principally, that here those practical lessons of disease might be given, which are of such moment in constituting students of medicine accurate judges of morbid actions, and of the best means for their removal. We live in a practical age, and all idle and visionary speculations are fast fading away, in the dim twilight of neglect and oblivion.

The professors of the Cincinnati College strive to be effective practical teachers of medicine—they wish no higher glory to attach to their names, should they be fortunate enough to be enrolled among the sons of light in the history of our science, than that they successfully labored to elevate medicine among the certain sciences, and to confer upon it a higher and more diversified capability of practical usefulness.

The voice of instruction within the walls of our school, we wish to be echoed within the wards of this hospital. The principles inculcated there, we desire to be exemplified here. What the lips of the lecturer there teaches, we hope the more eloquent appeals of nature will here confirm and re-enforce.

The object of therapeutics, is the discovery of the true indications of cure in diseases—the object of materia medica, is to select suitable remedies for the fulfilment of such indications. By indication of cure is to be understood, the end or final point to which we direct our remedial measures. Thus the indication of cure in a recent incised wound with clean edges, is to produce re-union by the first intention; and to attain this end, the lips of the wound are brought, and kept in a state of coaptation. And so likewise we say, that the indication of cure in pleurisy is to relieve the pulmonary organs of the embarrassment in their great function of respiration, arising from the inflammation of their pleural investment, by a subdual of the anormal action going on there. This we accomplish by the energetic employment of the lancet, by tartar emetic, and counter irritation.

But an important question meets us at the very threshold of this discussion—which is this: Does nature to any just extent, and with any degree of intelligible language, impart to us a sound knowledge of curative indications? In other words, before the physician interferes at all in disease, should he wait to see the evolutions of nature's efforts, or should he at once, after a careful scrutiny of the case, take the matter in his own hands?

This is a question of such practical consequence, that I hope to have your most dispassionate and yet earnest attention to the remarks about to be submitted on it.

You often hear nature magnified and glorified for her recuperative powers. *Natura morborum medicatrix*—nature is the physician that I trust to, says one. Nature must be our guide in sickness, and we must do nothing contrary to her indications, says another. We must leave much to nature, adds a third. Well, gentlemen, let us see how far these propositions will carry us, were we to follow their light. If nature is a real, medicating, restorative agent, endued with a skilful power to cure, then, let us abandon physick, and not harass our patients by multiplying officiously unnecessary aids.

The very fact, that a doctor of medicine is called in, is abundant proof that nature is not adequate to the recovery of the patient. Well! but nature, though not alone sufficient to cure, should be consulted before we prescribe. But how consulted? as a medical adviser, or as a witness. Most certainly the latter. What constitutes the essential character of disease, as contradistinguished from health? Disease is the usurped control, or ascendant influence, of the inherent tendency of all organized bodies to change their form—in other words, in proportion to the severity of the morbid process, will be the diminution of the authority and dominion of nature. By nature is meant the laws of the economy, to preserve the normal, physiological condition of the body. We are not now speaking of the capability of these laws, within certain limits, of restoring the system to health, after disease is arrested. Nor do we now bring up for discussion, the question of the ability of the spontaneous actions of life, to rid the organism of disease. The question now under consideration is, whether nature furnishes us any available or valuable indications of cure in disease? I answer, she does not. There is not a single morbid phenomenon which, fairly interpreted, the physician can trust as a faithful indication, to a sound practice at the bed side. Take gastritis as an example—here we have an irritable stomach—with an urgent tendency often to vomiting;—well, do we obey the indication presented by this gastric irritability, and urge on the emesis by our remedies? Assuredly not. Take another example. Here lies a patient, ill with apoplexy; what indication does nature here present, as physician to her own oppressed state, capable of being made available to her relief? Not one. We must therefore consult nature, not in her medicating interferences, but merely in her signals and demonstrations of disordered action;—not look on her with the hopeful eye of one who expects her to hold up her finger to us, to guide us in the true path of practice, but scrutinize her symptoms with the eye of a friend, who, with earnest regard, desires so to interpret her signs and evidences of derangement, as to ascertain how, to what extent, and in what localities, she is oppressed; and then devise and execute plans of relief and restoration.

Medicine is from beginning to end, an artificial scheme of restoring the sick. The sick have no desire for medicines, they nauseate them, they take them by constraint, and not by any suggestions of the organs, nor from any indications which the patient is enabled to discover for their employment. Careful observations, patient trials, and logical deductions, must be employed most assiduously to make a good practitioner. It is worse than idle, nay it is very mischievous to the cause of practical medicine, for us to rely in any degree upon the medicating powers of nature, as affording any sound ground-work for curative indications.

Does the vitality of the blood afford any legitimate therapeutic indications? No. Then why, with fond and illusive earnestness, speculate about the supposititious, inherent, independent vital endowment of the blood? Why, with a fervid fancy, heat our minds in the chase of a nonentity? The appearances of the blood drawn in disease are too ambiguous to be relied on, as affording indications of cure. There are few, very few enlightened physicians of the day, who place any firm reliance on the manifestations of the blood in the vessel, in which it has been drawn by the lancet, as indicative of the nature and seat of a disease. The sizy, and cupped states of the blood in connexion with the symptoms and results of our remedies, may conduce to a just diagnosis; but no exclusive reliance can be safely reposed in any appearance of the blood, after being deposited in the vessel. The size of the orifice, the shape and size of the vessel receiving the blood, and even the distance which the stream traverses before it passes into the vessel, essentially modify the aspect of that fluid.

How far can the Humoral Pathology assist us, in the detection of the true indications of cure in disease. Does this antique, venerable and much venerated system of pathology, throw any light on the true therapeutical indications by which scientific medicine is to be governed at the bed side? Here is the field on which this time-hallowed and hoary-headed theory, or rather hypothesis, should achieve its most signal triumphs. But alas for its advocates! It deserts them at the bed side; and at the very moment when its illustrations and enforcement are most anxiously anticipated, like the fabled hero of the Æneid, it escapes in a cloud from the scene of action, and leaves its votary to seek for other aids and better lights to guide him.

There is not an intelligent practitioner in modern times, who seriously adverts to these morbid matters of the blood for the indications of treatment in disease. Much is said, aye, and said often most plausibly and eloquently, about the probabilities of the blood being contaminated, but we read of no certain proofs being given on the point. There are no chemical analyses appealed to, by which we may have verified and authenticated such suppositions; there are no distinct positive announcements made, that by taking a little blood from a patient's arm in fever, or any other disease, we can ascertain by its taste, color, acid or alkaline nature, the nature and seat of his complaint. There is not a single indication of cure, that we can build on these supposed morbid states of the blood. We never seriously advert to morbific matters floating in the blood during life, as affording us any direction how to proceed in our plan of remedial measures. And after death, what medical man is there, however impregnated his faculties may be with these humors of fancy, who in sober trial, ever makes an appeal to humoralism, to impart information to his mind, in reference to a just pathology of the malady which destroyed the patient.

Diagnosis is the basis of all scientific medicine at the bed side. To establish a just diagnosis, we must have certain land-marks, by which our clinical inquiries are to be guided. These land-marks are, 1st. disordered sensations, 2d. disordered functions, and 3d. disordered structure. Or they may be expressed by the terms lesions of innervation, lesions of function, and lesions of structure.

Disease is denoted by one, or all, of these departures from a normal or physiological state, and may be generalized under the heads of Irritation, Inflammation, and Fever. To ascertain that our patient is ill, and the extent or intensity of that illness, we first make inquiries into his own sensations; secondly, investigate the state of the various functions of the body, beginning at the head, thence descending to the thoracic and abdominal viscera, and terminating our inquiry by a reference to the condition of the skin. In this inquiry, we, in a special manner, refer to the circulatory and nervous systems; and by a strict examination of the pulse, the state of the tongue, the decumbitus, etc., endeavor to arrive at correct conclusions respecting the constitutional, as well as local, disturbances, which the disease may have set up, or from which it may have originated.

In all our clinical perquisition, we must ever strive to attain precise views relative to the kind, as well as locality of morbid processes. The theory of Rush and Broussais, that disease is a unit, is contradicted at every step of our investigations at the bed side. The study of clinical medicine would be comprised in two propositions, were this theory of the unity of morbid action correct: 1st. Increased action, 2d. Diminished action. Hence but two indications would guide us—to diminish action where it is augmented, and to augment it where it is diminished. And the practice of medicine, as legitimately grounded on such a pathological conception, would comprise the lancet in one hand, and the brandy bottle in the other.

We determine the deviations from healthy action in the system, by an accurate inspection of the symptoms, as well as by the history of the seizure, and the effects of remedies which may have been employed.

Signs and symptoms are not the same. A sign is the index of a thing, and admits of only one interpretation. Thus we say, and say correctly, that the rise or fall of the mercury in the thermometer, or barometer, is a sign of the increase or diminution of the heat or weight, of the atmosphere.

A board with a certain direction on it, at a particular turn of the road, we call a sign-board—there it stands, pointing one way, telling us one plain thing. But a symptom may be, or it may not be, a true sign of a certain morbid affection. To become a true or legitimate sign of a morbid process going on in the animal economy, a symptom must have the light of a lucid and accurate interpretation thrown upon it, by the investigating mind. Symptoms are often ambiguous—they deal with us in a double sense, and unless a due degree of wariness attend the clinical examiner—unless one symptom is construed by the reflected light of another symptom—we shall often commit sad and serious mistakes in our pathological inferences. Take but one example. Here, you see one man affected with severe pain in the stomach, with jactitation, and cold clammy skin;—there, is another patient with gastric pain equally severe, and a restlessness as great; but in the one case, there is a comparatively calm, equable, fluent pulse; in the other, there is a small, contracted, hard, incompressible, quick pulse. Here the pulse is denotive of the essential difference of the two cases—one of slight indisposition, perhaps gastrodynia, or indigestion; the other a severe and dangerous gastritis.

Besides the symptomatology, we are often aided in our clinical inquiries, by the physical signs of disease in the thoracic, abdominal and pelvic regions. The genius of a Corvisart, and a Laennec, has brought into requisition and successful employment, anscultation, as an important diagnostic means for the elucidation of many an enigma in clinical medicine;—percussion, palpation, and anscultation, mediate and direct, have opened to our minds a hitherto much neglected source of examination in many obscure maladies of the heart, lungs, and abdominal and pelvic viscera.

We have determined that our patient is ill—1st. By his sensations; 2d. By the deviations of functions, and 3d. By the aberrations of structure, through the symptoms and physical signs. We next determine what the *nature* of his complaint is; whether it is irritation, inflammation, or fever. We afterwards determine whether the case is primarily referable to a constitutional, or to a local origin; and upon these different but coincident elements of a just and enlightened conception of the patient's state, we establish our indications of treatment.

To exemplify—take a case of neuralgia. Here we have only the declarations of the patient to guide us in the ascertainment of his sufferings; but we can, by a careful scrutiny into the case, find out, perhaps, how the general system is at fault, and how the habits of life, of the patient, may modify the existing condition of things, and vary our plan of treatment. Neuralgia may exist, nay most frequently does exist, without inflammation. Sometimes it is dependent on some local irritant, such as a tumor pressing on the nerve, or a spicula of bone, or the remnant of a fine needle, irritating the nerve. At times there exists a gastric disturbance, or hepatic derangement, or irregularity of the uterine function; but often there seems no local origin of the affection.

There are many practitioners, since the publication of the work of Teale, who regard neuralgic complaints as having an exclusive origin in the spinal cord. We read and hear a great deal about spinal irritation; but this pathology is too indefinite; it is only, indeed, a modified form of the old doctrine of the nervous system being the source of the disease. We know this without any parade of discussion about the matter; the nervous system being affected, whether in the spinal or cerebral portions, or in the distributions of it, the question is, *why* is it, and *how* is it affected? But these questions I shall resume in the lecture which will be devoted to the discussion of neuralgia.

Diseases differ in their nature, as well as points or sources of origin, and modes of termination. There is such a thing as irritation, distinct from inflammation; and there is a pathological state entitled fever, distinct from both irritation and inflammation, though frequently partaking of both of these conditions, or modes of abnormal action. But neither is irritation, inflammation, nor fever, a unit. Irritation varies with the cause originating it, with the seat it occupies, with the constitutional peculiarities, the age, sex, and other modifying circumstances; and so does inflammation, and likewise fever.

To erect judicious indications of cure on the ratio symptomatum, we must, then, ascertain the nature, and seat of the disease. Is it irritation, or is it inflammation, or is it fever? If irritation, or inflammation, how is the constitution affected? Is it deeply implicated in the local complaint, or does the local complaint depend upon the state of the system? What is the state of the circulation? What the condition of the liver, bowels, kidneys, and skin, as respects their secretory products? What the condition of the general apparatus of innervation, and of the digestive functions? These and other points we must ascertain. In all our prescriptions, let us ever, with watchful eye, advert to the state of the secretions; for from this quarter we shall gather a pretty sure diagnosis of our patient's danger. To restore the secretions is a great leading indication in disease. Blood-letting, emetics, cathartics, nauseants, narcotics, etc., are often but preliminary means to the re-establishment of the secretions.

Another leading indication, often presented to the judgment of the skilful practioner of medicine is, to derive action from the organ undergoing a morbid process, to another part—hence the benefits often resulting from what is termed revulsive medication. In an enlarged view, we might fairly consider emetics, cathartics, nauseants, diuretics, diaphoretics, etc., as all, in a greater or less degree, subserving a useful curative end, by their revulsive powers. But among the more technical counter-irritants, or revulsives, we place blisters, setons, cauteries, etc. We often employ one class of internal remedies with this indication in view—purgatives are often brought into requisition when we wish to derive action from the head, or from the joints, in the inflammatory affections which seize upon those parts. In chronic complaints of a local character, revulsive medication is too much neglected. A permanent discharge kept up by a seton, or issue, will do more in many local inflammations, than all other means of cure. In dropsy, we often transfer action from one portion of the capillary system to another. Our diuretics act by deriving action from the exhalent capillaries of the cellular texture, or of the peritoneum, to the kidneys; and thus, by an arrest being put to the increased secretion of the part originating the hydropic effusion, the absorbents pursuing their regular function, the fluid is taken up.

Another indication of a leading kind, which frequently demands our medical interference, is that of direct depletion. The great agent for the cure of acute inflammation is the lancet. This powerful remedy, is often brought into active employment. Bloodletting, general and local, purgatives, low diet, nauseants, emetics, cathartics, these are our great means for the fulfilment of this leading indication of depletion, or debilitation. This indication is more frequently our guide in disease than any other single indication. To deplete judiciously is the main purpose of treatment in numerous maladies. The Italians talk of the contra stimulant property of tartar emetic; but I attribute its virtues as a remedy in pneumonitis and other phlegmasiæ, to its alterant rather than its contra stimulant power.

Another leading indication, which sometimes draws upon our remedial resources, is debility:—stimulants and tonics, are however, much abused medicines; and debility, in the majority of instances, is nothing but a symptom of inflammation, irritation, or fever; and in consequence, is to be incidentally treated by a strict reference to the cause originating it. Debility has been elevated into a commanding position, in the etiological scale of some medical authorities. Debility of the heart, gives rise to congestion, say they, of the portal circle; and this, in its turn, plays a most conspicuous part, in all the morbid phenomena witnessed. Consistently with this theory, all remedies which further debilitate the action of the heart, should increase the abdominal congestion, and thus by our depletory plans—the only generally successful plans in febrile affections, and inflammations—we should but increase and perpetuate disease, instead of curing it.

Here we see a palpable contradiction between speculation and experience; and therefore debility should not be regarded, in the vast majority of diseases, as any thing more than a symptom of morbid action; for in proportion to the intensity of the functional disturbance, and structural lesion, will be the degree of debility.

An additional indication of treatment, is frequently brought to light in the medical procedure to be had in irritation, inflammation, or fever—which is an alterant, or alterative plan of treatment. Now, we call that medicine an alterant, which, without any direct depletion, stimulation, or any other immediate obvious result of agency on the nervous, circulatory, or secretory apparatus, subverts morbid action, and restores health. Mercury is our great alterant; yet mercury may, and often does, incidentally, excite the heart's action, or depress the powers of life, as the case may be; and almost uniformly, when its alterant operation is of a benign restorative character, promotes the secretions. And yet the superior curative agency of mercury is not owing to any one, or all of these accompaniments, or consequences, of its constitutional action, but to its peculiar alterant powers. When judiciously administered, in very minute doses, it produces its beneficent results with no appreciable direct interference with the nervous, circulatory, or secretory apparatus. At one period—for instance, in the days of Sydenham, it was universally thought by medical practitioners, that the remedial efficacy of mercury was owing to its immense capability of increasing glandular action; and hence, when a patient was placed under a course of mercury, his mouth was made very sore, and the saliva by pints was made to flow from his mouth, from day to day, till all the morbific matter in the blood had escaped. The rapid manner in which mercury checks the inflammatory process, and arrests the deposition of coagulable lymph, as for instance in a case of iritis, demonstrably evinces, that it has some peculiar controlling influence over the capillary system.

There are many, very many cases of disease, which call for the curative agency of alterants. We have mercury as the great anti-inflammatory, anti-febrile alterant—we have arsenic, sarsaparilla, nitric acid, and iodine, as alterants endowed with characteristic properties variant from those possessed by mercury. And lastly, often there springs up in the progress, or is presented from the commencemeut of a complaint, an indication for the administration of a peculiar set of remedies, which act in quieting the disturbed actions of the nervous system. These we denominate anodynes; and a very important and useful set of medicines they are in numerous cases of curable, as well as incurable diseases. Often is it of the last moment to the recovery of our patient. that sleep shall be procured. And as often is it essential to the successful issue of our plan of treatment, that urgent pain be subdued or alleviated, and the irritability of the system be calmed by the interposition of some narcotic remedy. Opium stands pre-eminently distinguished as a soother of pain—as the *magnum dei donum*—the great gift of God, to remove, or mitigate the sufferings in many maladies, to which our mortal frames are subject.

Pain is often but a symptom of inflammation; and of course the indication in inflammatory affections is not to attempt the deadening of pain by anodynes, but to cure the inflammation, and then remove the pain, if it should continue.

Pain is likewise often the result of disturbed or suspended function—thus we have intense uterine pain in dysmenorrhœa;—here anodynes are called for, to alleviate the sufferings of the patient, though we do not remove the cause, the functional irregularity of the organ, by their exhibition. Suspended, or disturbed function in one organ, often induces pain in another. Thus cephalalgia is often produced by the torpid state of the bowels;—here a purgative will be the best anodyne.

After active depletion by the lancet in inflammatory attacks an anodyne is often of sovereign efficacy in quieting the system, and diffusing an equable excitement to the great relief of the suffering organ. Anodynes are often indicated as corrigents of other medicines.

These, then, are our indications of cure in disease:—1st. The promotion of healthy secretions; 2d. The derivation of action; 3d. Reduction of the activity of the powers of life; 4th. The invigoration of the vital energies; 5th. The alteration of capillary action; and 6th. The subdual, or mitigation of pain and irritability,

But in the wise government to be exercised over the diversified modes in which irritation, inflammation, and fever affect the powers of the organism, and the endless ways in which they interchangeably run into, and reciprocally influence each other, we are often called on to adjust our prescriptions by a conjunction of two, three, or even more of the different articles, which operate to the fulfilment of our indications.

Irritation, as I shall hereafter endeavor to prove, is the primal link in inflammation; but irritation may exist in some of its forms or phases, without inflammation; though inflammation cannot obtain without the antecedent occurrence of irritation.

Fever, as I hope to prove to you hereafter, may exist as an idiopathic affection, though fever cannot exist many hours without the danger of awakening inflammation in some vital portion of the system. And this tendency of fever to the production of inflammation, has given origin to the various theories of the local origin of fever, which, from time to time, have been so popular in the profession.

I earnestly recommend the subject of therapeutic indications to your zealous attention. It is a subject of great practical consideration. Sydenham seems to have deeply felt the momentous importance of the questions involved in the establishment of true curative indications; for, says that sagacious practitioner, "the practice of physic chiefly consists in being able to discover the true curative indications," And in another place he says "whoever considers the matter thoroughly, will find that the principal defect in the practice of physic, proceeds, not from a scarcity of medicines to answer particular intentions, but from the want of knowing the intentions to be answered."

And Boerhave, in the preface to his Aphorisms, remarks, that "it is the very characteristic of a good physician to make a true indication."

Without a knowledge of indications, the physician becomes a mere unscientific administrator of drugs.

